# Laparoscopic Management of Gallbladder Duplication: A Case Report

**DOI:** 10.7759/cureus.10675

**Published:** 2020-09-27

**Authors:** Ali H Alsharedah, Sarah M Alotaibi, Basim F Khan, Askar K Alshaibani, Saad A Algarni

**Affiliations:** 1 Family and Community Medicine, Imam Abdulrahman Bin Faisal University, Dammam, SAU

**Keywords:** gallbladder duplication, laparoscopy, acute cholecystitis

## Abstract

Laparoscopic cholecystectomy has become one of the most commonly performed abdominal surgeries worldwide. Several anatomic variations and congenital malformations in the gallbladder and biliary anatomy have been described. We reported the case of a middle-aged woman who presented with jaundice and abdominal pain. Her laboratory investigations revealed an elevated bilirubin level (mainly the direct component). The patient showed an improvement in the clinical and laboratory parameters after conservative management. Then, the patient was prepared for a laparoscopic cholecystectomy which revealed a duplication of the gallbladder with two distinct cystic ducts draining independently to the common bile duct. The procedure was completed uneventfully. This type of gallbladder duplication is among the least common types. The case highlights the importance of having a meticulous intraoperative evaluation of the biliary anatomy to avoid potential complications and injuries.

## Introduction

The gallbladder is a pear-shaped hollow viscus that serves to store the bile produced by the liver and concentrates it to be released after meals [1]. It is formed from the caudal part of the foregut at six weeks of gestation [2]. Anatomic variations and congenital malformations of the gallbladder are well-recognized. However, they are rare and could be part of some syndromes [3]. The awareness of these variations is of paramount importance because of their direct clinical and surgical implications.

Gallstone disease is very common worldwide and it is considered the most common indication for abdominal surgical procedures in the United States [4]. Gallstones develop when there is an imbalance in the composition of the bile resulting in crystallized deposits of bilirubin and cholesterol [5]. Herein, we describe a case of gallbladder duplication that was incidentally identified during laparoscopic surgery for acute cholecystitis.

## Case presentation

A 54-year-old woman presented to the emergency department with a four-day history of right upper quadrant abdominal pain. She described the pain as constant and sharp in nature. The pain radiated to the right shoulder and was associated with nausea and two episodes of vomiting. She reported having previous similar episodes of this pain over the previous year that occur after fatty meals but the pain was milder and had spontaneous resolution. The patient noticed slight yellowish discoloration of her skin and eyes. The patient noticed a dark urine and pale stool. In addition, he recently developed a generalized pruritus. Her past medical history is remarkable for a long-standing gastroesophageal reflux disease which is well-controlled with proton-pump inhibitors. Of note, the patient does not drink alcohol and does not have any history of recreational drug use. There was no history of recent travel.

Upon presentation, she was icteric and her pulse rate, blood pressure, temperature, and respiratory rate were observed to be 112 beats per minute, 124/68 mmHg, 37.9° C, and 13 breaths per minute, respectively. The abdominal examination revealed a tenderness in the right upper quadrant with a positive Murphy sign. There were no clinical signs of peritonitis and no stigmata of liver disease were observed. Her basic hematological and biochemical investigations revealed a hemoglobin level of 13.5 g/dL, a leucocyte of 12.0 × 10^3^/μL with a left shift (85% neutrophils), a total bilirubin of 2.1 mg/dL with a direct bilirubin of 1.2 mg/dL, a gamma glutamyl transferase of 200 U/L, and her levels of urea and electrolytes were normal. The urinalysis revealed no abnormalities. An ultrasonography of the abdomen was further performed to confirm the diagnosis of obstructive jaundice. A dilated common bile duct (8 mm) with the presence of a small stone was observed. Pericholecystic fluid and several gallbladder stones with acoustic shadow were found.

**Figure 1:**
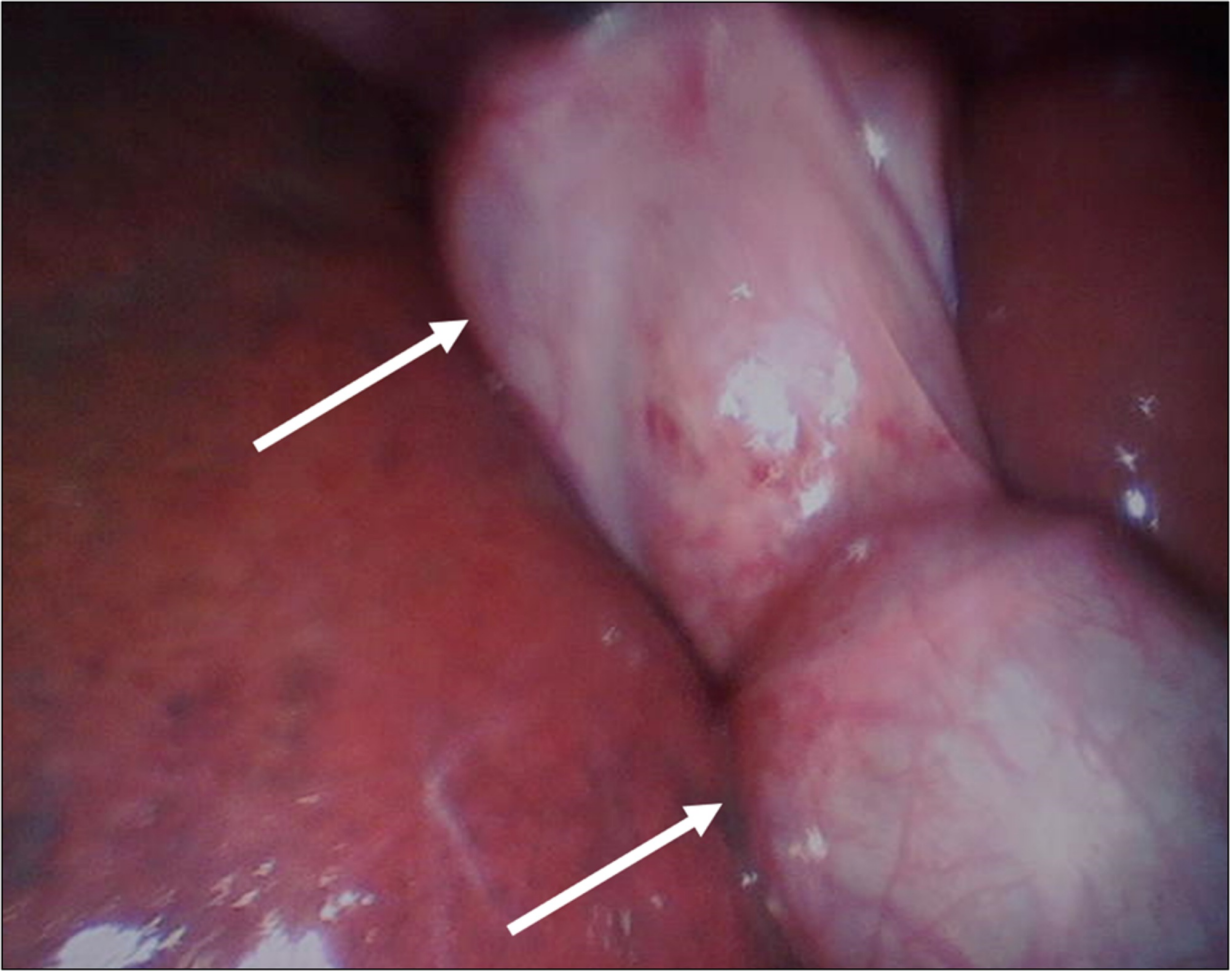
Laparoscopic View Laparoscopic view demonstrating the presence of two gallbladders (arrows).

Given the aforementioned clinical and imaging findings, the patient was started on conservative treatment and intravenous ceftriaxone 1 g daily. She demonstrated clinical and laboratory improvement within 48 hours. The patient was prepared for a laparoscopic cholecystectomy. The surgery was performed under general anesthesia and four ports were inserted to carry out the procedure. A diagnostic exploration was performed after establishing the pneumoperitoneum and introducing the trocars. It revealed the presence of a gallbladder duplication (Figure 1).

**Figure 2:**
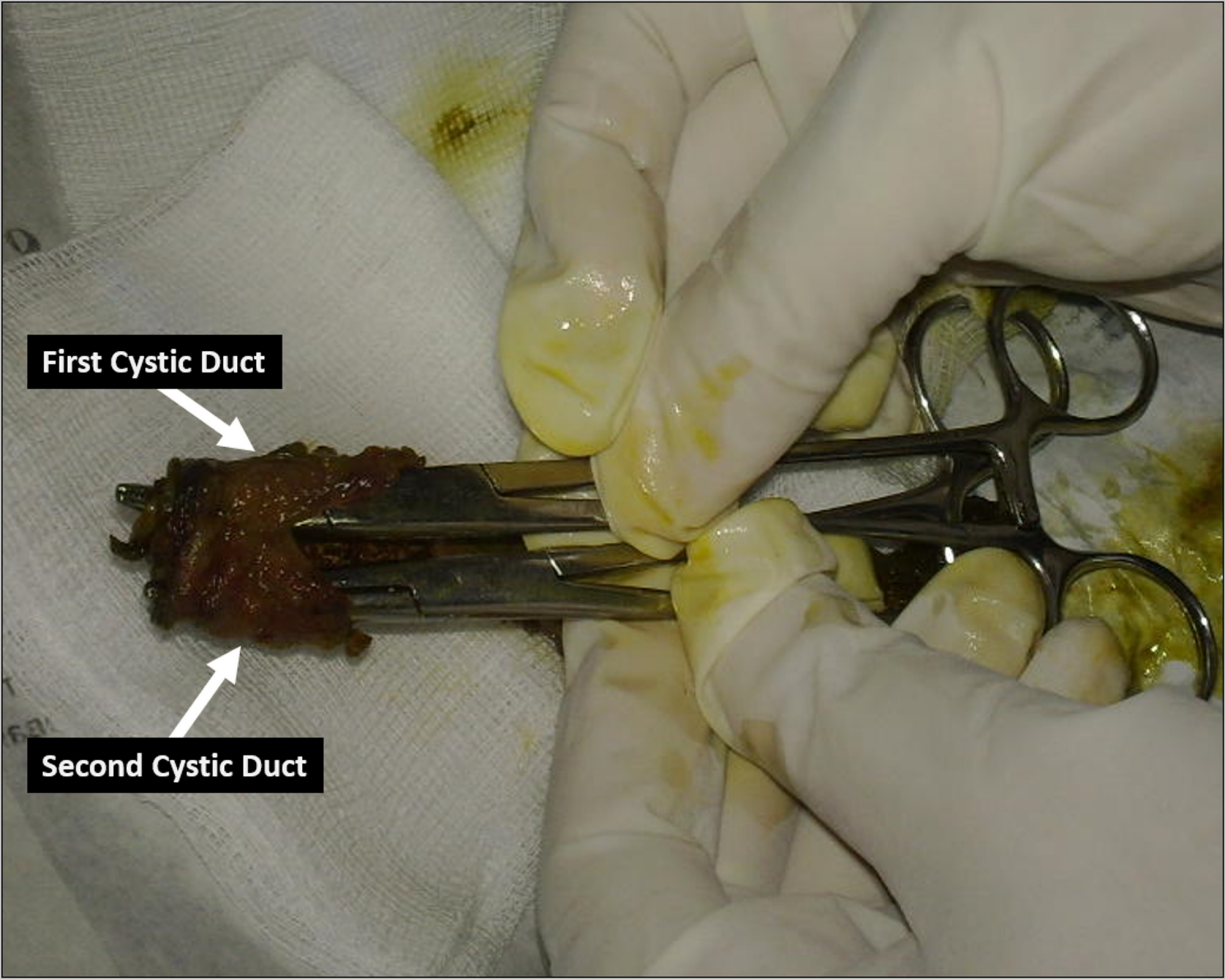
Resected Specimen A gross image for the resected specimen of gallbladders showing two distinct cystic ducts (arrows).

Meticulous dissection was performed. The cystic artery and both cystic ducts were ligated. Both gallbladders and their cystic ducts were resected (Figure 2).

Intra-operative cholangiogram showed no evidence of choledocholithiasis. The total operation time was 90 minutes and the estimated blood loss was 50 ml. The patient tolerated the procedure well and was discharged on the fourth postoperative day. She remained symptom-free at one-month follow-up.

## Discussion

We described the case of laparoscopic cholecystectomy in a patient with a gallbladder duplication. Gallbladder duplication is an unusual congenital anomaly with an estimated incidence of 1 per 5000 individuals [6]. Several classification systems have been proposed for a gallbladder supplication [7]. In Boyden classification, there are two main types which are bilobed gallbladder (vesica fellea divisa) and true duplication (vesica fella duplex) which is further classified into Y-shaped that has two cystic ducts uniting before entering the common bile duct while the H-shaped type has two cystic ducts entering the common bile ducts independently, as in our case, and is considered the most common type [8]. Harlaftis classification is a newer classification for gallbladder duplication that is based on embryological development and has two types [9]. In type 1 (split primordial group), there is a single cystic duct and this type is further subdivided into septated, V-shaped, and Y-shaped. In type 2, there are two distinct cystic ducts.

There are no unique presenting clinical features of gallbladder duplication. Each gallbladder may be involved in the pathologies that can affect the single gallbladder such as acute cholecystitis. Gallbladder duplication is usually discovered intraoperatively, as in our case, and could lead to surgical difficulties, including conversion to laparotomy and the risk of biliary injuries [10]. It is essential to recognize this condition intraoperatively since missing this condition could require the patient to have repeated surgery for the other gallbladder. It should be noted that it is recommended to resect both gallbladders even if the pathology is in one of them due to the risk of recurrence so that the patient does not need to undergo another operation for the second gallbladder [11].

Our patient responded with the conservative treatment and did not need to undergo further investigations. In cases of obstructive jaundice with failure to respond with conservative treatment, advanced imaging technique, including endoscopic retrograde cholangiopancreatography, magnetic resonance cholangiopancreatography, could be used to delineate the biliary anatomy and diagnose the duplication of gallbladder preoperatively. While the patient had undergone ultrasonography on presentation in the present case, this modality is largely user-dependent and gallbladder variations may not be recognized. The differential diagnosis of gallbladder duplication on ultrasound includes choledochal cyst, gallbladder fold, gallbladder diverticulum, and intraperitoneal fibrous bands [12].

## Conclusions

Gallbladder duplication is a rare congenital anomaly. Careful evaluation of the vascular and biliary anatomy is crucial to avoid intraoperative complications, including common bile duct injuries. Laparoscopic surgery is a feasible option in the management of a gallbladder duplication with gallbladder disease.

## References

[1] Nagral S (2005). Anatomy relevant to cholecystectomy. J Minim Access Surg.

[2] Kamath BM, Piccoli DA (2003). Heritable disorders of the bile ducts. Gastroenterol Clin North Am.

[3] Senecail B, Texier F, Kergastel I, Patin-Philippe L (2000). Anatomic variability and congenital anomalies of the gallbladder: ultrasonographic study of 1823 patients (Article in French). Morphologie.

[4] Comitalo JB (2012). Laparoscopic cholecystectomy and newer techniques of gallbladder removal. JSLS.

[5] Schirmer BD, Winters KL, Edlich RF (2005). Cholelithiasis and cholecystitis. J Long Term Eff Med Implants.

[6] Boyden EA (1926). The accessory gall‐bladder - an embryological and comparative study of aberrant biliary vesicles occurring in man and the domestic mammals. Am J Anat.

[7] Khandelwal RG, Reddy TV, Balachandar TG, Palaniswamy KR, Reddy PK (2010). Symptomatic “H” type duplex gallbladder. JSLS.

[8] Vezakis A, Pantiora E, Giannoulopoulos D, Fontara S, Kontis E, Polydorou A, Fragulidis G (2019). A duplicated gallbladder in a patient presenting with acute cholangitis. A case study and a literature review. Ann Hepatol.

[9] Harlaftis N, Gray SW, Skandalakis JE (1977). Multiple gallbladders. Surg Gynecol Obstet.

[10] Guajardo-Salinas GE, Martinez-Ugarte ML, Abourjaily G (2010). The use of intraoperative cholangiogram during laparoscopic double cholecystectomy. J Surg Case Rep.

[11] Goh YM, Goh YL, Ewan LC, Turner PD, Lapsia S, Subar DA (2015). A case report of duplex gallbladder and review of the literature. Int J Surg Case Rep.

[12] Goiney RC, Schoenecker SA, Cyr DR, Shuman WP, Peters MJ, Cooperberg PL (1985). Sonography of gallbladder duplication and differential considerations. AJR Am J Roentgenol.

